# Germination and seedling growth of *Calluna vulgaris* is sensitive to regional climate, heathland succession, and drought

**DOI:** 10.1002/ece3.10199

**Published:** 2023-07-04

**Authors:** Kristine Birkeli, Ragnhild Gya, Siri Vatsø Haugum, Liv Guri Velle, Vigdis Vandvik

**Affiliations:** ^1^ Department of Biological Sciences University of Bergen Bergen Norway; ^2^ Bjerknes Center for Climate Research Bergen Norway; ^3^ The Heathland Centre Alver Norway; ^4^ Møreforsking Ålesund Norway

**Keywords:** allocation, coastal heathlands, drought experiment, dwarf‐shrub heath, functional traits, germination rate, plasticity, population ecology, secondary succession, seedling growth, water potential

## Abstract

The coastal heathlands of Northwest Europe are highly valued cultural landscapes, that are critically endangered due to land use and climatic changes, such as increased frequency and severity of drought events. Our study is the first to assess how the germination and early seedling growth of *Calluna vulgaris* respond to drought. In a factorial design field experiment, we exposed maternal plants to three in‐situ drought treatments (control, 60%, 90% roof coverage), across three successional stages after fire (pioneer, building, mature), and two regions (60°N, 65°N). Seeds from 540 plants within the experiment were, weighed, and exposed to five water potentials, ranging from −0.25 to −1.7 MPa, in a growth chamber experiment. We recorded germination (percentage, rate), seedling growth (above‐ vs. belowground allocation), and seedling functional traits (specific leaf area [SLA], specific root length [SRL]). Overall variation in germination between regions, successional stages, and maternal drought treatments was largely mediated by variation in seed mass. Plants from the northernmost region had higher seed mass and germination percentages. This is indicative of higher investment in seeds, likely linked to the populations' absence of vegetative root sprouting. Seeds from the mature successional stage germinated to lower final percentages than those from earlier successional stages, especially when the maternal plants had been exposed to drought (60% and 90% roof coverage). Exposure to reduced water availability decreased germination percentage and increased the time to 50% germination. Seedlings fully developed in the range −0.25 to −0.7 MPa, with increased root:shoot and lower SRL during reduced water availability, suggesting a resource‐conservative response to drought during the early stages of development. Our results thus suggest a sensitivity to drought during the germination and seedling life‐history stages that may reduce *Calluna's* ability to re‐establish from seeds as the incidence and severity of droughts are projected to increase under future climates.

## 1 INTRODUCTION

Human‐induced climate change has caused a global temperature rise of 1.1°C over the last century, a trend that will continue unless drastic reductions in carbon emissions are made (IPCC, 2023). This global warming drives changes in weather systems, including an increase in more extreme weather events, such as drought (Mann et al., 2017; Stott, 2016). In oceanic boreal regions, such as the west coast of Norway, climate change is thus projected to lead to an overall increase in precipitation; but also an increase in the frequency and severity of drought events (Skaland et al., 2019). These extreme weather events add ecological stress to already vulnerable ecosystems (Gonzalez et al., 2010).

Until about a decade ago, the most commonly observed ecological response to global warming in the Arctic was increased shrub growth, known as “arctic greening” (IPCC, 2014). In recent years, however, a new phenomenon, “arctic browning,” is becoming increasingly prevalent through the arctic and boreal zones globally (Bjerke et al., 2017; Phoenix & Bjerke, 2016; Treharne et al., 2019; Wang & Friedl, 2019). This refers to the widespread die‐back of evergreen dwarf‐shrub heath vegetation, and while the underlying causality is not fully resolved (Myers‐Smith et al., 2020), the phenomenon has emerged in parallel with frequency of extreme weather events (Phoenix & Bjerke, 2016). Arctic browning has thus been linked to increased environmental stress due to episodes of low precipitation, especially during winter months, reducing snow coverage and exposing the evergreen vegetation to frost and drought (Bjerke et al., 2014, 2017). Such winter drought‐driven browning events have also been observed at subarctic latitudes, especially in the anthropogenic coastal heathlands of Northwest Europe (Phoenix & Bjerke, 2016).

Coastal heathlands dominated by evergreen dwarf shrubs, notably the keystone species *Calluna vulgaris* (L.) Hull (hereafter referred to as *Calluna*), are a characteristic and valuable cultural landscape found throughout the oceanic regions of Europe, with a history dating back more than 5000 years (Birks et al., 1988; European Commission, 2008; IPBES, 2018; Kaland, 1986). Coastal heathlands are currently red‐listed throughout their range due to the abandonment of traditional low‐intensity land use (Hovstad et al., 2018; IPBES, 2018; Janssen et al., 2016; Lindgaard & Henriksen, 2011; Wilson et al., 2019) and are now further threatened by climate change. Ecosystem responses to drought have been documented in coastal and lowland heathlands (Britton et al., 2001; Haugum et al., 2021; Log et al., 2017), including a link between “browning” and drought‐induced reduction in primary production and reproduction of *Calluna* (Phoenix & Bjerke, 2016).

In a traditionally managed coastal heathland system, prescribed burning is used to create a fine‐scale mosaic of heathlands in different successional stages after fire; pioneer, building, and mature (de Hullu & Gimingham, 1984; Kaland, 1986). This successional mosaic provides year‐round access to quality pasture for free‐ranging livestock (mostly sheep, but also goats, horses, cattle), while also increasing biodiversity (Velle et al., 2014). Recent research suggests that heathland management may also lower the risk of large‐scale drought damage (Haugum, 2021), due to age‐related physiological variation in the dominant dwarf shrub, where mature *Calluna* is more negatively affected by drought than building or pioneer‐stage plants (Haugum et al., 2021). This suggests that abandonment of the traditional grazing and burning practices may render heathlands more vulnerable to drought damage, and thus increase browning.

*Calluna* revegetates after fire both vegetatively and from seed (Mallik & Gimingham, 1985; Mallik et al., 1984; Mohamed & Gimingham, 1970), with variation due to succession and ontogeny. While all successional stages after fire have abundant seed production (Mallik et al., 1984), older *Calluna* plants, in the mature stage and beyond, have lower probability of producing root sprouts after fire (Berdowski & Siepel, 1988; Calvo et al., 2002; Hobbs & Gimingham, 1984; Meyer‐Grünefeldt et al., 2015; Miller & Miles, 1970). This suggests that later‐successional *Calluna* stands are more dependent on regenerating from seed for regeneration. Both very young (Meyer‐Grünefeldt et al., 2015, 2016) and older mature (Haugum et al., 2021) *Calluna* plants have been shown to exhibit lower resistance to drought than intermediately aged plants. These findings suggest the plant's investment in seeds and seedling recruitment success under drought may vary with the ontogeny and over succession (see Table 1 for a summary of predictions).

**TABLE 1:** Overview of predictions (*P*) for seed mass, germination percentage and niche, and seedling traits including specific leaf area, specific root length, and root:shoot ratio for each of the parameters:population, successional stage and maternal drought treatment, also including across all comparisons.

	Population	Successional stage	Maternal drought treatment	Across all comparisons
Seed mass	P1: The northern population will have a greater seed mass as an adaptation to the lacking vegetative regeneration	P2a: The mature successional stage will have a higher seed mass because of the reduced ability to resprout vegetatively P2b: Seeds from the mature successional stage with drought‐exposed maternal generation will have a lower seed mass because of the mature stands' sensitivity to drought	P3: Drought‐exposed maternal generation will have a greater seed mass as a plastic response to the drought treatment in the field	P4: A greater seed mass across all factors will increase germination percentage and increase root: shoot ratio as a greater seed mass is linked to a higher germination percentage and more drought‐adapted traits
Germination strategy (germination percentage and niche)	P5: The northern population will have a higher germination percentage as an adaptation to the lack of vegetative regeneration P6: The southern population will have a broader germination niche as an adaptation to historically more drought‐exposed habitat	P7: The mature successional stage will have a lower germination percentage and smaller germination niche because of lower seed quality	P8: Drought‐exposed maternal generation will have an increased germination percentage and broader germination niche due to a plastic response to drought in the maternal plant	–
Traits (root: shoot, SLA, and SRL)	P9: The southern population will have a higher root:shoot ratio, lower SLA, and greater SRL in response to drought as it is more adapted to periods of drought	P10: Mature successional stages will have a lower root:shoot ratio, greater SLA, and lower SRL due to its sensitivity to drought	P11: Drought‐exposed maternal generation will result in a higher root:shoot ratio, lower SLA, and greater SRL as a plastic response	P12: Across all factors there will be an increase in root:shoot and SRL, and a decrease in SLA as a response to drought

*Note*: All predictions are based on the predicted responses to reduced water availability during germination and early seedling growth. Broad germination niche refers to germination under a wide range of drought conditions in the lab.

*Calluna* is a widespread species that exhibits local adaptations to both climate and traditional land‐use management (Spindelböck et al., 2013; Vandvik et al., 2014). Several lines of evidence indicate possible geographic variation in drought responses. First, populations of *Calluna* throughout the southern European heathlands do respond differently to drought (Ibe et al., 2020; Meyer‐Grünefeldt et al., 2016). More southern populations exhibit a greater drought tolerance, while Atlantic populations are more sensitive (Ibe et al., 2020). No study to date has tested for differences in *Calluna* drought responses in northern Atlantic heathlands, including in the northernmost distributional range where arctic browning is now occurring. Second, populations along the south–north climatic gradient in Norway have been shown to differ in regeneration modes and responses, with northern *Calluna* populations having higher temperature requirements for seed germination and lacking vegetative resprouting, compared to southern populations, which have lower germination temperature requirements and use both seeds and root sprouting for regeneration (Nilsen et al., 2005; Spindelböck et al., 2013). The lack of vegetative resprouting in the northern populations might predict a higher investment in seeds compared to the southern populations, as seeds are the only mode of recruitment here. At the same time, the southern populations are adapted to a warmer climate, which historically has exposed them to more frequent drought (Meteorologisk Institutt, 2021), suggesting higher past selective pressure for drought tolerance. Third, there are broad‐scale geographic patterns in the phylogenetic structure of *Calluna* across Europe, with Northern and Southern populations in Norway being of different descent (Durka et al., unpublished). This suggests a potential for local adaptations, including adaptations to drought, across the northern parts of the *Calluna* range. These lines of evidence might infer differences in seed germination responses to drought between Northern and Southern populations, again with potentially contrasting predictions resulting from the northern populations' higher dependence on seeds for recruitment, and the southern populations' adaptations to a warmer climate (Table 1).

Drought can induce a plastic response in the maternal generation during seed formation that could influence seed germination behavior and success (Donohue & Schmitt, 1998; Mayer & Poljakoff‐Mayber, 1982). An increasing incidence and severity of drought due to climate change begs the question of whether such responses could occur in *Calluna*. During drought, plants tend to produce larger seeds as a stress response, which is positively correlated with seedling survival during drought (Gianoli & González‐Teuber, 2005; Lloret et al., 1999; Vera, 1997). Seed mass is also positively correlated with a higher root:shoot ratio (Gianoli & González‐Teuber, 2005; Lloret et al., 1999), which is beneficial during drought (Karcher et al., 2008; Xu et al., 2015). *Calluna* is sensitive to drought during the seedling stage because of the lower root:shoot ratio of juvenile plants compared to building and mature stands of *Calluna* (Meyer‐Grünefeldt et al., 2015). If *Calluna* has the ability to show a plastic response to drought, differences in seed size and seedling drought responses could appear depending on the maternal drought exposure (Table 1).

Finally, seedlings may respond to drought in terms of biomass allocation and functional traits, for example by increasing root:shoot ratio, decreasing specific leaf area (SLA), and increasing specific root length (SRL) (Liu & Stützel, 2004; Metcalfe et al., 2008). Seed mass is a key mediating seedling recruitment success, and with increasing seed mass we generally expect increased germination percentage, broader germination niche, larger seedling size, and higher plasticity in the root:shoot ratio in response to drought conditions experienced by the seedlings. We, therefore, expect many of the responses outlined above to be mediated by seed mass (Table 1).

In summary, the literature outlined above suggest a series of partially contrasting predictions about the responses and tolerances of *Calluna* seed recruitment to drought, and how these might vary among populations, successional stages, and maternal drought treatments (Table 1).

In this study, we will investigate the drought responses of *Calluna* seed germination and seedling traits, and test the predictions outlined above about variation in seedling drought responses, by comparing responses to reduced water availability in the germination and seedling traits of seeds originating from northern and southern populations, from pioneer, building, and mature successional stages, and from control versus experimental drought climatic conditions. Using a germination experiment in the laboratory, we are asking the following three questions:

1. Do population origin, successional stage, and the maternal generation's exposure to drought affect seed mass in *Calluna*, and does greater seed mass infer greater germination percentage and drought tolerance?
2. Do population origin, successional stage, and the maternal generation's exposure to drought affect *Calluna* seed germination, and in particular the seed and seedling responses to reduced water availability?
3. Are population origin, successional stage, and maternal generation's exposure to drought effects evident beyond the seed germination stage, in the seedling traits?

To answer these questions, we use seeds collected from a drought experiment in the field where coastal *Calluna* heathlands are subjected to ambient conditions (the control plot) and drought treatment by rainout shelters covering 60% and 90% of the plots (see Haugum et al., 2021 for details). The experiment is replicated across two regions in Norway (ca. 60°N and 65°N), reflecting different climates and also different genetic origins (Durka et al., unpublished), and across three successional stages after prescribed fire: pioneer, building, and mature. The seeds from each of 540 maternal plants from the field experiment were germinated in five petri dishes with an agar growth medium manipulated to deliver different levels of water availability to achieve a five‐level water potential gradient (including a control). The experiment was conducted in a growth chamber, where seed germination timing and percentage were recorded over a 105‐day period. SLA, SRL, and aboveground and belowground biomass was measured on the emerging seedlings.

## 2 METHODS

### 2.1 Study species (*Calluna vulgaris* (L.) Hull)

*Calluna* is a monotypic genus distributed across all of Europe from Scandinavia to the Iberian Peninsula and from the Ural Mountains to the Azores (Tutin et al., 1973). It is an evergreen dwarf shrub typically 10–50 cm tall but can reach up to 1 m in standing height (Lid & Lid, 2017). Its flowering season is from July to September, when it flowers with numerous small purple flowers (Clapham et al., 1981), which are mainly pollinated by insects, such as bumblebees and other bees (Knuth, 1908), but also by wind (Mahy et al., 1998). Seeds are small, with a maximum diameter of 0.58 mm (SE = 0.016 mm) (Bullock & Clarke, 2000), allowing seed transport by wind (Beijerinck, 1940). The reproduction in *Calluna* is a combination of vegetative resprouting and seedling recruitment from soil seed storage, with both germination and resprouting strategies varying over the ontogeny and with successional age with lower resprouting in older stands (Berdowski & Siepel, 1988; Calvo et al., 2005; Miller & Miles, 1970), and throughout the species' range, with no resprouting in the northern populations (Nilsen et al., 2005; Spindelböck et al., 2013; Vandvik et al., 2014).

### 2.2 Site description

**FIGURE 1:**
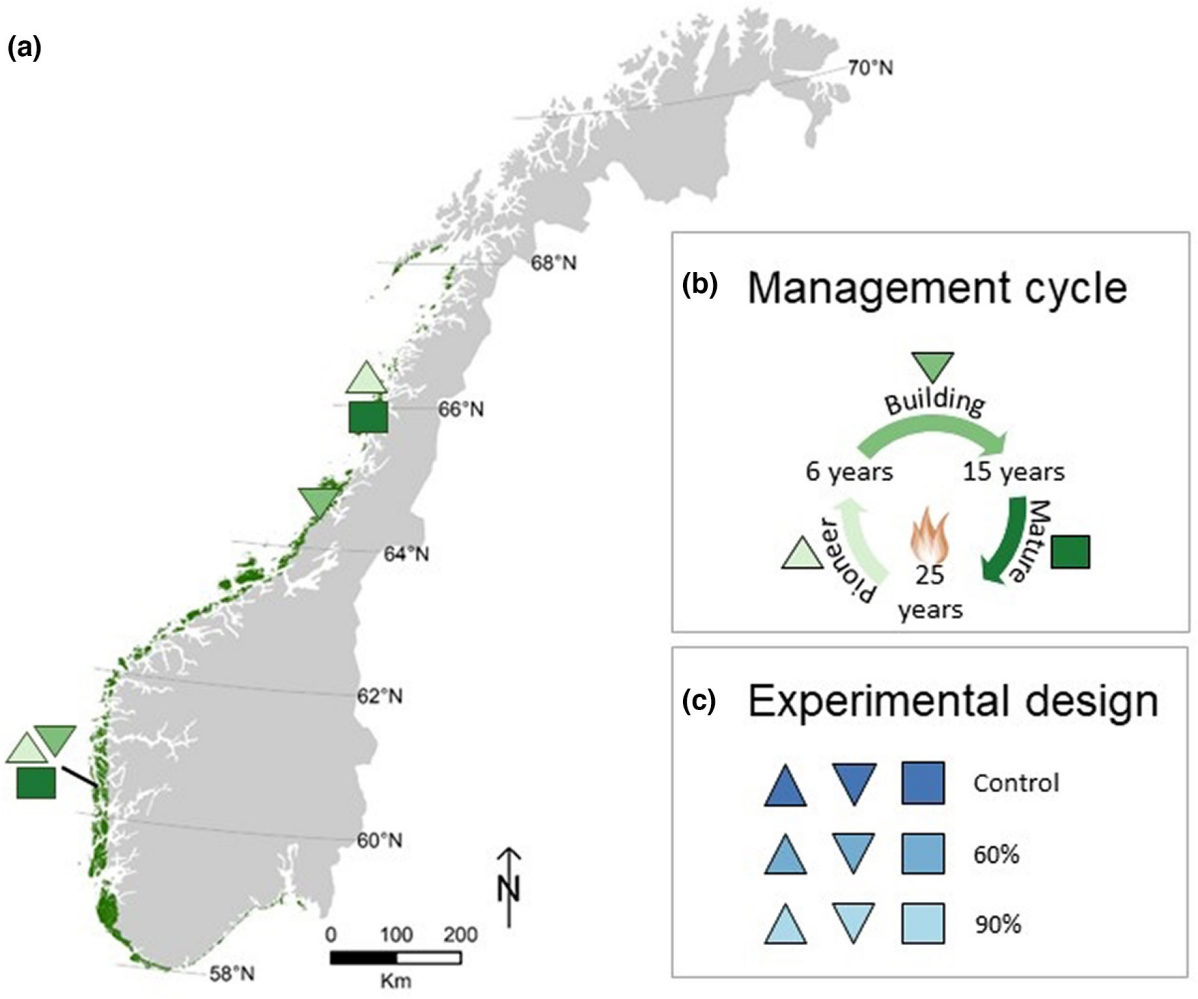
(a) Distribution of Atlantic heathlands in Norway, including study sites (southern sites are overlapping in the map). (b) Successional stages of the traditional management style (up triangle = pioneer stage, down triangle = building stage, square = mature stage). (c) The set‐up of the experimental design per site, with three replicates of the three parental drought treatments (control, 60%, 90%) for a total of 54 plots (2 populations × 3 successional stages × 3 parental drought treatments × 3 replicates).

The study was conducted in two bioclimatic regions, with three heathland successional stages sampled in each for a total of six sites. The northern sites are Buøya representing the pioneer stage, Haverøya the building stage, and Skotsvær the mature stage (Table 2, Figure 1). In the southern region, all three stages and hence sites are found on the same island, Lygra (Table 2). All six sites are extensively grazed by old norse sheep or spælsau (Haugum et al., 2021). All sites have an oceanic climate with heathland vegetation dominated by heathers, especially *Calluna*, and a land‐use history with fire and free‐range grazing. Most sites are acidic, wet to dry heathland, except for Buøya, which is a slightly more calcareous heathland (Halvorsen, 2016). Haverøya has warmer winters and higher precipitation compared to the other northern sites (see Table 2 for details), but due to a known lack of vegetative resprouting (Nilsen et al., 2005; Spindelböck et al., 2013) and genetic similarities to northern sites (Durka et al., unpublished), we expect the responses to be similar to other northern sites.

**TABLE 2:** Ecological and climatic site information.

Population	Site name	Successional stage	MAP (mm)	MST (°C)	MWT (°C)	Latitude (decimal degrees)	Longitude (decimal degrees)	Burn year	Total number of seeds collected
North	Store Buøya	Pioneer	1254 ± 184	13.4 ± 1.3	0.7 ± 1.6	65.83677	12.224506	2014	3147
North	Haverøya	Building	1720 ± 461	13.3 ± 1.4	1.5 ± 1.9	64.779	11.2193	2010	2966
North	Skotsvær	Mature	1254 ± 184	13.4 ± 1.3	0.7 ± 1.6	65.79602	12.22450	Before 1980	2217
South	Lygra 1	Pioneer	2020 ± 345	13.8 ± 1.5	3.4 ± 1.8	60.70084	5.092566	2013	3119
South	Lygra 2	Late building	2020 ± 345	13.8 ± 1.5	3.4 ± 1.8	60.70084	5.092566	2004	3012
South	Lygra 3	Mature	2020 ± 345	13.8 ± 1.5	3.4 ± 1.8	60.70084	5.092566	1996	1662

*Note*: Mean annual precipitation (MAP), mean summer temperature (MST) (June–August), and mean winter temperature (MWT) (December–February) are based on data from 1990 to 2019 (Haugum et al., 2021).

### 2.3 Maternal drought experiment

In the summer of 2016, three blocks of 70–200 m^2^ were set out in homogenous vegetation in each of the six sites. In each block, we installed three 2 × 2 m plots, selected to be dominated by *Calluna* while avoiding larger rocks, bare ground, and animal tracks. To avoid grazing in the experimental plots, each plot was fenced and had the roofs set up in spring 2017, except for Haverøya, which was fenced in spring 2018. Each plot was randomly assigned a drought treatment (control, 60% and 90% roof coverage), and rainout shelters were installed following the Drought‐Net protocol (Yahdjian & Sala, 2002), with three replicates of each drought level. The obtained reductions in rainfall were 32.1 ± 10.3% for 60% roof coverage and 43.5 ± 20.3% for the 90% roof coverage (Haugum et al., 2021).

### 2.4 Collecting seeds in the field

In each plot, 10 individuals of *Calluna* were tagged with a unique identifier and measured for functional traits annually from 2016 to 2019 (Haugum et al., 2021). The marked *Calluna* were selected to represent the full range of plant sizes within each plot (Haugum et al., 2021). From each of these individuals, we collected at least 50 seeds. Due to death or grazing disturbance before fences were up, extra individuals within the plot were collected as necessary to ensure 10 plants were sampled from each plot. All seed collection was undertaken between 30.09.2019 and 30.10.2019, which is at the end of the flowering season of *Calluna* in Norway (Lid & Lid, 2017). The seeds were dry stored in coffee filters in the lab for a year, with the assumption of little loss of viability as *Calluna* seeds from this region have a long life span in soil seed banks (Måren & Vandvik, 2009). The total number of seeds collected and used was 16,123.

### 2.5 Seed germination experiment

Of the 10 individuals collected above, three were randomly chosen to be used in a germination experiment to assess germination and seedling responses to reduced water availability in *Calluna*. Due to some missing individuals and cases of low seed production, 6864 seeds were sown in 726 petri dishes with the following design and replication:

2latitudes×3successional stages×3maternal drought treatments×3replicate plots×3individual plants×5levels of reduced water potentials×10seedsperpetri dish

resulting in a replication of *n* = 9 maternal plants per site, stage, and treatment in the field experiment, with the unit of observation being the individual petri dish.

Reduced water availability conditions were established using agar infused with a polyethylene glycol (PEG, molecular weight 8000; 191 Sigma) solution, with five levels of drought, including a control, reaching from −0.25 to −1.7 megapascal (MPa) referred to as levels WP1–WP5 with increasing level of drought. The preparation of the dishes followed the protocol based on Van der Weele et al. (2000) as adapted by Gya et al. (2023). Specifically, 1% agar was autoclaved to avoid fungal or bacterial growth during germination, then 20 mL of 1% agar was added to 90 mm petri dishes. The PEG was added after the agar solidified to avoid polymerization of the agar. PEG was dissolved in distilled water to reach the target water potentials (Table 3) with 30 mL of PEG solution added to the 20 mL agar dishes. The dishes were covered by parafilm and set to equilibrate for 4 days. Before adding seeds, excess liquid solution in the petri dishes was carefully removed.

**TABLE 3:** Water potential level (referred to as WP1–WP5 throughout) and final water potentials of the different water availability treatments used in the germination experiment.

Water potential level	Final water potential for petri dishes (MPa)	Grams of solid PEG added per liter media for overlay PEG solution
WP1	−0.25	0
WP2	−0.5	250
WP3	−0.7	400
WP4	−1.2	550
WP5	−1.7	700

*Note*: 30 mL of polyethylene glycol (PEG) solutions were added to 90 mm petri dishes containing 20 mL 1% agar to achieve different final water potentials (MPa) in the dishes.

After preparing the petri dishes as described above, 10 seeds from each individual plant were placed in each of five petri dishes, one for each water availability treatment. For individuals with less than 50 seeds, treatments were prioritized in the following order: extreme drought (WP5, −1.7 MPa), control (WP1, −0.25 MPa) followed by WP3, WP2, and WP4. Within each petri dish, seeds were placed apart from each other on the agar surface using tweezers and gloves to avoid contamination. Petri dishes were covered with parafilm to avoid drying and placed in a growth chamber (Sanyo Incubator MIR‐553) at 18°C (Grimstad, 1985), with the light cycle set at 8 h light and 16 h darkness, the optimal germination conditions for *Calluna* seeds from our regions (Måren & Vandvik, 2009).

The seeds were monitored once a week until 14 days after onset, then observations were done twice a week until day 29. Monitoring frequency returned to once a week when the cumulative germination curves started flattening. The last observations were made on day 105 after onset.

### 2.6 Trait measurements

Before the germination experiment, the seeds from each of the 10 individual (10–50 seeds) were weighed collectively, and the mean seed mass per individual was calculated by dividing the bulked weight by the number of seeds. This was done because of the low seed mass of *Calluna*. We report air‐dried seed mass (not oven dried) as the seeds were to be used in the germination trial.

Germination traits for analyses included germination percentage, which is the germinated seeds as a percentage of number of seeds in a dish, *T*_50_ (time to 50% germination), and germination duration (number of days to reach final germination percentage). To test for viability, all seeds that did not germinate in the control (WP1) were tested using an embryo integrity (squish) test and cut test (Halbritter et al., 2020) and viability was calculated as the sum of germinated plus viable seeds as a proportion of total number of seeds in the petri dish. We assume that the resulting viability is constant across the maternal individuals' petri dishes, as seeds were randomly assigned to water availability treatments from the same maternal plant.

To measure seedling growth and allocation, we did a subsampling of one seedling for each petri dish and measured for the following traits: SLA – specific leaf area, SRL – specific root length, total biomass, and allocation to belowground.

The chosen individual was harvested 1 week after recorded emergence, with the aboveground (hypocotyl) and belowground (epicotyl) sections separated at the point where the individual emerged from the agar. The belowground section was weighed and the length (mm) of the single, unbranched root was measured. The aboveground parts were divided into leaves and stems, all parts were individually weighed and the leaves scanned using CanoScan LiDE 220. All seedling parts were then placed in separate coffee filters tagged with the seedling ID in a drying oven at 60°C for 48 h (Díaz et al., 2016). After drying the biomass was again weighed. SLA was found using the scanned area of the upside of the leaf divided by its dry mass and SRL was found using the length of the root divided by its dry mass (Díaz et al., 2016). To calculate the total leaf area, the “LeafArea” package in R was used (Katabuchi, 2019). The belowground allocation was calculated as the ratio between belowground and total biomass.

### 2.7 Statistical analysis

To investigate the effect of population, successional stage, maternal drought treatment, and water availability on *Calluna* germination percentage we used a generalized linear mixed effect model and for count data and seedling traits we used a linear mixed effect model, performed in R (R Core Team, 2019) using the lme4 package (Bates et al., 2020) and lmerTest (Kuznetsova et al., 2020). A binomial distribution was assumed for germination (1 = germinated and 0 = ungerminated), which was analyzed using a logit link function. The count data, *T*_50_ and time to max germination, and the traits, SLA, SRL, and root:shoot ratio, were normally distributed after log transformation, while the seed mass was normally distributed without transformation. For all these variables we assumed Gaussian distributions. For each response variable (seed mass, germination percentage, *T*_50_ (time to 50% germinated), maximum germination, SLA, SRL, and root:shoot ratio) we constructed one global model to evaluate the overall treatment effects and response patterns. In these models, the fixed effects included the population, successional stage, maternal drought treatment, water availability, and all interactions. Note that water availability was not relevant for the model for seed mass, and so was not included here. We also constructed additional models with seed mass as an additional explanatory variable for germination percentage and root:shoot ratio, to test specific predictions (see below). In all models, nested random effects include site (Lygra 1–3, Store Buøya, Haverøya, Skotsvær), plot (each replicate of all drought treatments per site), and ID (Identification number of the plant collected per plot). The predictions outlined in Table 1 were then tested by inspecting specific effects or interactions in these full models, and by using post‐hoc tests from the package “emmeans” (Russell, 2021) to further explore differences between specific levels of the predictor variables and interactions, according to the specific predictions as outlined below.

Questions and their predictions were investigated as follows:

1. Do population origin, successional stage, and the maternal generation's exposure to drought affect seed mass in *Calluna*, and does greater seed mass infer greater germination percentage and drought tolerance?

P1, P2a, and P3 predict population, successional stage, and maternal drought treatment, respectively, to have significant effects on seed mass. We tested this in the models for seed mass by inspecting the population, successional stage, and parental drought treatment fixed effects. For P2b we also investigated the interaction between successional stage and maternal drought treatment within the same global model. P4 predicts that a larger seed mass across all factors (populations, successional stage, and parental drought treatment) will increase germination percentage, and increase root:shoot ratio. To test this, we used the residuals from a model testing the effect of seed mass for these responses (described above) and inspected the differences for the residual model and the global models described below.

2 Do population origin, successional stage, and the maternal generation's exposure to drought affect *Calluna* seed germination, and in particular the seed and seedling responses to reduced water availability?

As described in Table 1, P5 predicts a significant population effect on germination percentage. And similarly, P7 and P8 predict significant effects of fixed factors for successional stage and maternal drought treatment, respectively, which we inspected using the general model and error structure described above. P6 predicts a significant interaction between population and the reduced water availability treatments in the lab experiment. Interactive effects indicating changes in germination across WP treatments were also tested for P7 and P8, focusing on the successional stage and WP interaction in P7, and maternal drought treatment and WP interactions in P8.

3 Are population origin, successional stage, and maternal generation's exposure to drought effects evident beyond the seed germination stage, in the seedling traits?

P9, P10, and P11 were tested by inspecting the population, successional stage, and maternal drought fixed effects in models for SLA, SRL, and root:shoot ratio. Lastly, P12 was tested by inspecting higher‐order interactions involving reduced water availability in the full models for SLA, SRL, and root:shoot ratio.

## 3 RESULTS

### 3.1 Seed mass

**FIGURE 2:**
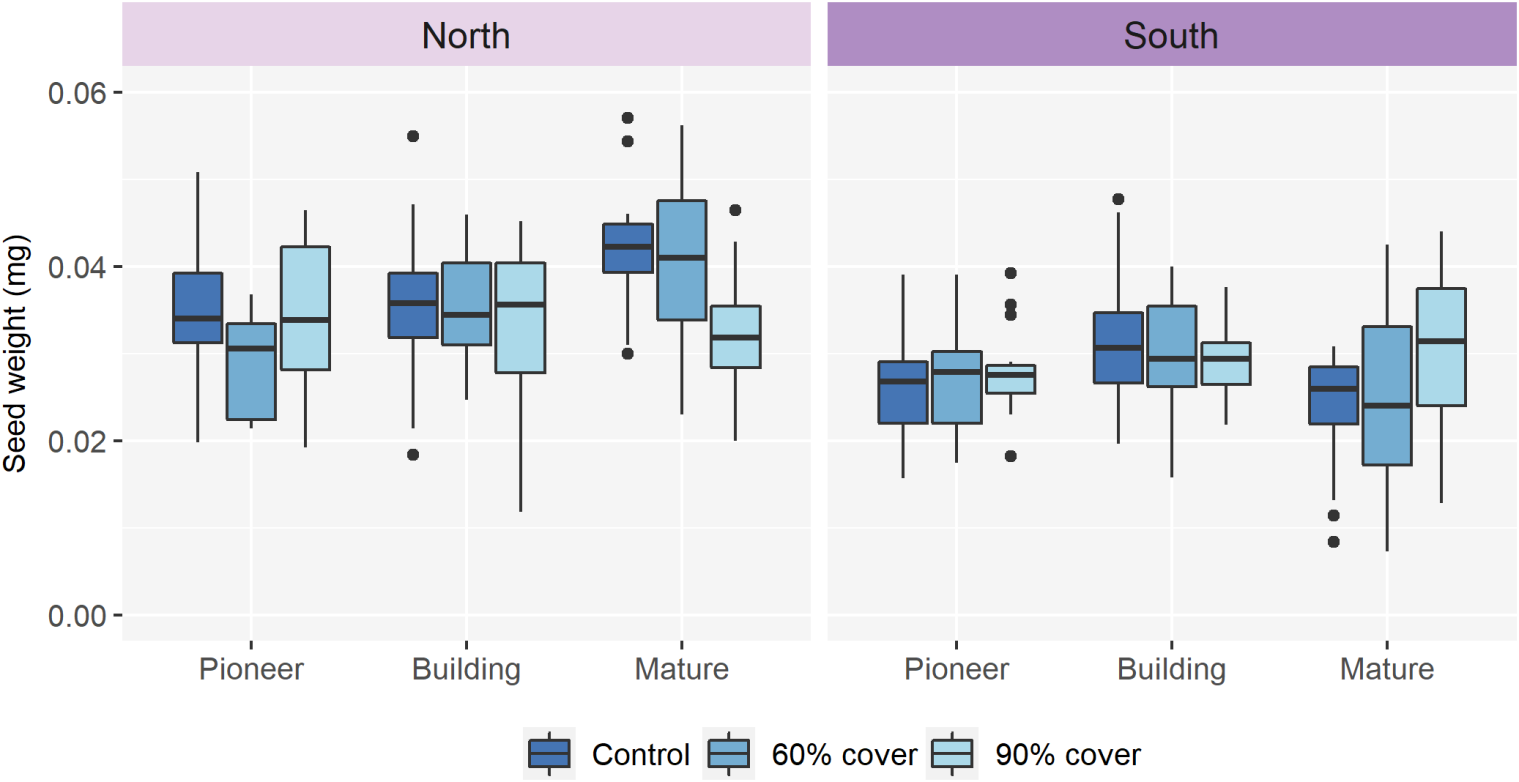
Seed mass (mg) of *Calluna* from the northern and southern populations, across three successional stages (pioneer, building, mature) and three levels of maternal drought treatment (control, 60%, 90% roof cover). The weight is calculated on a per seed basis, based on samples of 50 seeds from each of the 7 individuals sampled within the 54 field plots, except in cases where the individual plant had a low seed count. *N* = 16,123 seeds, 365 seed batches.

Seeds from the northern population are heavier than those from the south, averaging 0.035 ± 0.008 mg versus 0.028 ± 0.007 mg, respectively (Figure 2, Table A1, post‐hoc test north vs. south: *p* = .066). Mean seed mass does not vary over the succession within each region (post‐hoc test; pioneer vs. building and mature: *p* > .83 for both). Maternal drought affects seed mass, but only in the later‐successional stages, and with contrasting responses. Specifically, in the northern population, seed mass decreases in mature‐stage maternal plants exposed to drought (Figure 2, post‐hoc test; subset north; mature stage; control vs. 60% and 60% vs. 90% roof coverage: *p* = .0047 and *p* = .0065, respectively). In the southern population, in contrast, seed mass increases in the mature stage when the maternal plants are exposed to drought (Figure 2, post‐hoc test; subset south; mature stage; control vs. 90% roof coverage; *p* = .031).

### 3.2 Germination percentage and niche

#### 3.2.1 Germination percentage

**FIGURE 3:**
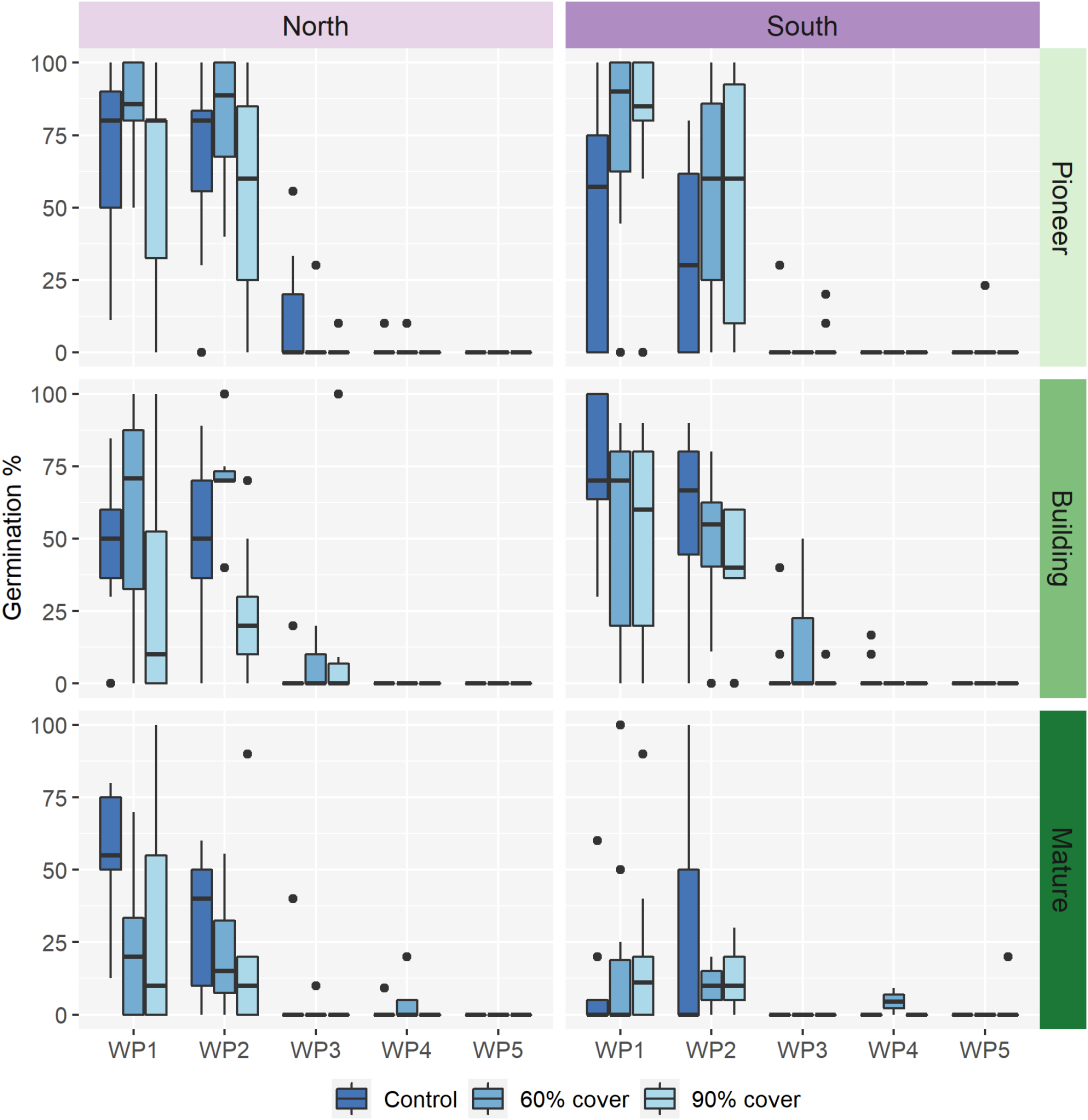
Germination percentage for the population in the north and south, across three successional stages (pioneer, building, mature), three maternal drought treatments (control, 60% and 90% roof coverage) and five levels of reducing water potentials (− 0.25 to −1.75 MPa). Data is collected during a 105‐day germination experiment, during controlled environments with 8 h growth light and 18°C. *N* = 6864 seeds, 726 petri dishes.

Reducing water availability has a major negative impact on seed germination, and this effect is moderated by population, successional stage, and the maternal drought treatment (Figure 3, Table A2). Out of the 6864 seeds sown, 5746 (83.7%) were viable and 1399 (20.4%) germinated across all water potentials. WP1 and WP2 make up 52.9% and 42.2% of the germinated seeds, respectively, and are not significantly different from each other (Figure 3, post‐hoc test, WP1 vs. WP2: *p* = .996). WP3, WP4, and WP5 all germinate to significantly lower percentages than the control (Figure 3, post‐hoc test: *p* < .001 for all contrasts mentioned), with WP3 only contributing 3.8% of the total germination, and WP4 and WP5 combined contributing 1.1%.

The northern population has a tendency toward higher germination compared to the southern population (Figure 3, Table A2). This effect is significant within WP2, (post‐hoc test; subset WP2; north vs. south: *p* = .0039). For WP1, patterns are more complex. Firstly, the higher germination in the north is evident in the mature stage and outside rainout shelters only (i.e., in the absence of maternal drought) (Figure 3, post‐hoc test; subset mature; maternal drought control; WP1; north vs. south: *p* = .0019). In the southern pioneer stage, germination increases in response to the maternal drought treatment, (Figure 3, post‐hoc test; subset south; pioneer; WP1; control vs. 60% and 90% roof coverage: *p* = .0604 and .0008, respectively).

The mature successional stage has an overall lower germination percentage compared to the pioneer and building stages (Figure 3, post‐hoc test pioneer vs. mature: *p* < .001; building vs. mature: *p* = .056, respectively). The mature successional stage is less tolerant of maternal drought, with germination percentages decreasing more in response to 60% and 90% roof coverage compared to the drought treatments in the pioneer stage (Figure 3, post‐hoc test; subset 60% and 90% roof coverage; WP1; Pioneer vs. Mature: *p* < .03).

#### 3.2.2 Time to 50% and maximum germination

**FIGURE 4:**
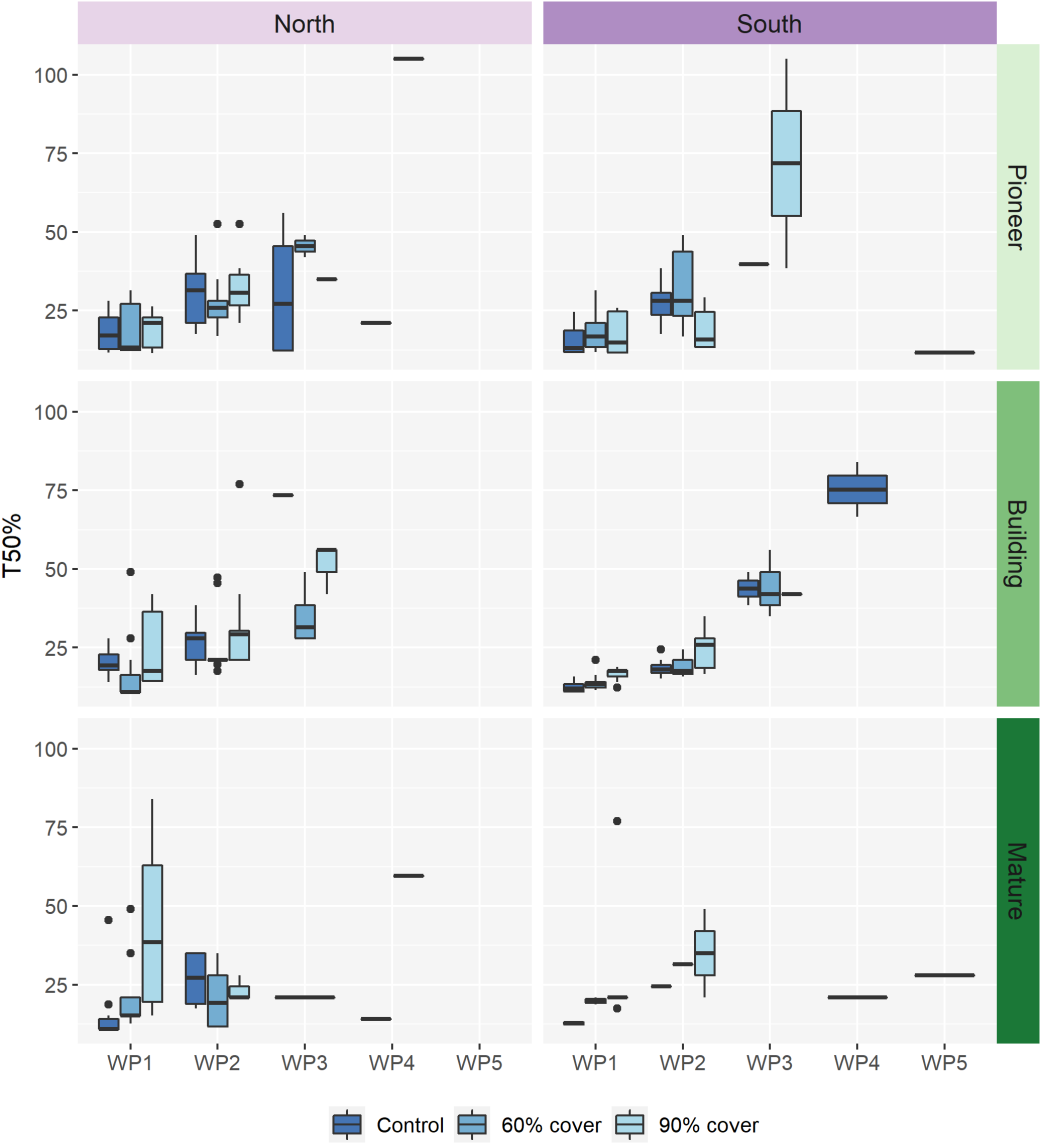
Time to 50% germination (days since sowing) across the five levels of water potential (−0.25 to −1.75 MPa) for the populations (north, south), successional stages (pioneer, building, mature), and three maternal drought treatments (control, 60% and 90% roof coverage). The data are based on weekly observations during the drought experiment done at 18°C with 8 h of growth light. *N* = 6864 seeds, 726 petri dishes.

Decreasing the water availability during germination reduces the germination rate, seeds in the WP1 treatment reached 50% germination after an average of 19 days, whereas seeds from WP2, WP3 and WP4 did not reach 50% germination until after 27, 45, and 53 days on average, respectively (Figure 4). These delays in germination within WP2 and WP3 are significantly different from the control (WP1) (Figure 4, *p* < .005, for both), while WP4 is non‐significant, but with a low sample size (*n* = 8, *p* > .4).

Within WP1 the northern population germinates slower than the southern population (Figure 4, Table A3, post‐hoc test subset WP1; north vs. south: *p* = .063). In the pioneer and building successional stages, germination is delayed with reduced water potential, while no difference between water potentials were found within the mature stage (Figure 4, Table A3, post‐hoc tests; subset pioneer; WP1 vs. WP2: *p* < .001. Post‐hoc test; subset building; WP1 vs WP2 and WP3, and WP2 vs. WP3: *p* < .001, for all. Post‐hoc test; subset mature; WP1 vs. WP2: *p* = .96), Seeds from maternal plants from the control and 50% roof coverage had their germination delayed with reduced water potential, while no difference in germination time was found when the maternal plant was exposed to 90% roof coverage (Figure 4, Table A3, post‐hoc test subset; control; WP1 vs. WP2: *p* < .001. post‐hoc test subset, 60% roof coverage, WP1 vs. WP2: *p* < .0024. Post‐hoc test; subset 90% roof coverage; WP1 vs. WP2: *p* = .44). *T*_max_ follows the same general patterns as *T*_50_ (Figure A2).

### 3.3 Seedling traits

#### 3.3.1 Specific root length, root:shoot ratio, and specific leaf area

**FIGURE 5:**
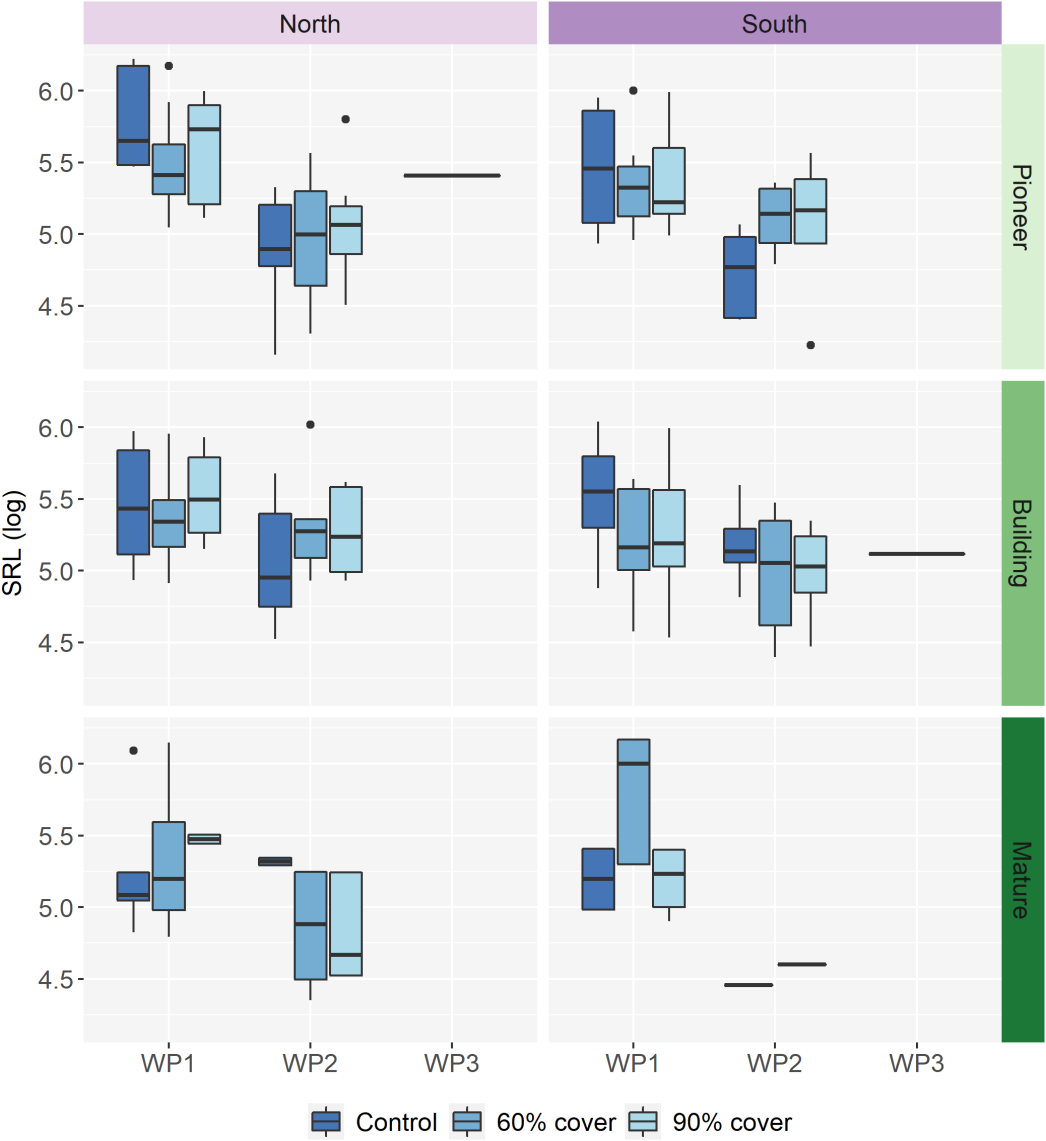
The logarithmic specific root length (SRL) for seedlings emerged in the control (WP1), the mild drought (WP2) and the medium drought (WP3). The data is based on one seedling for each of the petri dishes that had emerged seedlings. Seedlings were harvested for the length and weight measurements 1 week after registered emergence. *N* = 206 seeds, 206 petri dishes.

**FIGURE A1:**
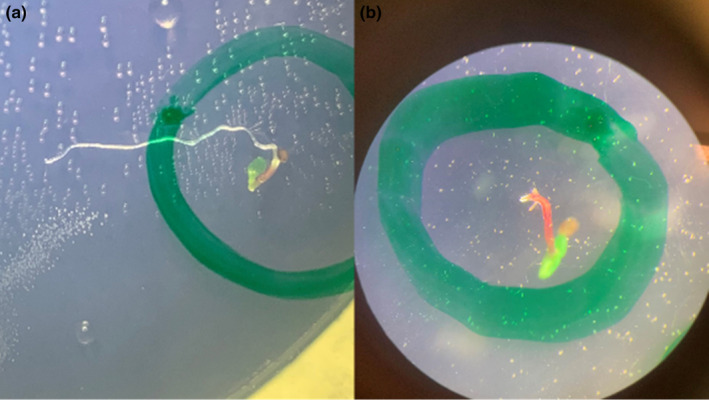
Seedlings emerging from the control (WP1) (a) and the mild drought (WP2) (b), at the day of harvest, 1 week after registered seedling emergence.

**FIGURE A2:**
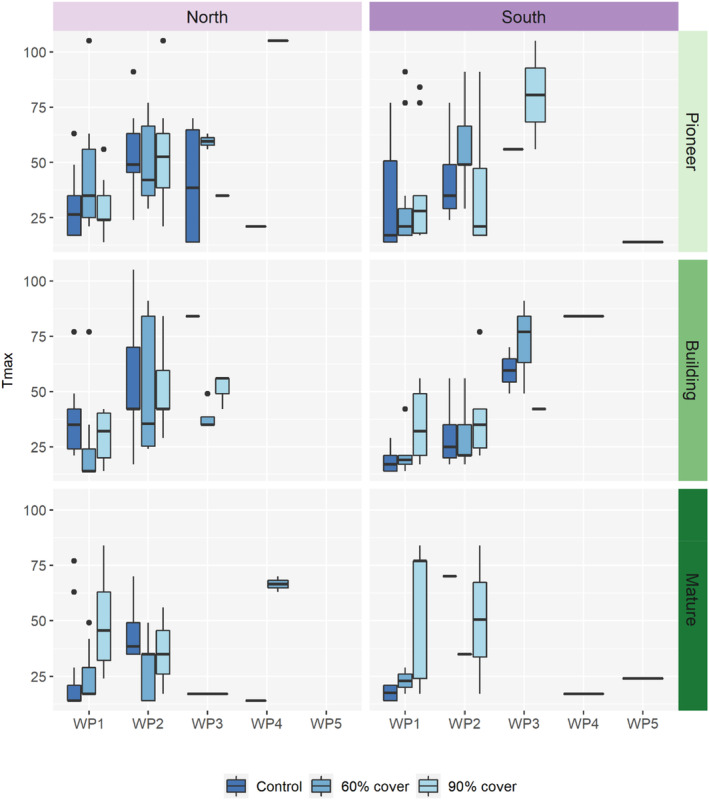
Time to maximum germination across the five levels of reducing water potentials (−0.25 to −1.75 MPa) for the populations (north, south), successional stages (pioneer, building, mature), and three maternal drought treatments (control, 60% and 90% roof coverage). The time across the *y*‐axis reaches from the day of sowing (*T*_0_) until the last day of observations on day 105. The data are based on weekly observations during the drought experiment done at 18°C with 8 h of growth light. *N* = 6865 seeds, 726 petri dishes.

**FIGURE A3:**
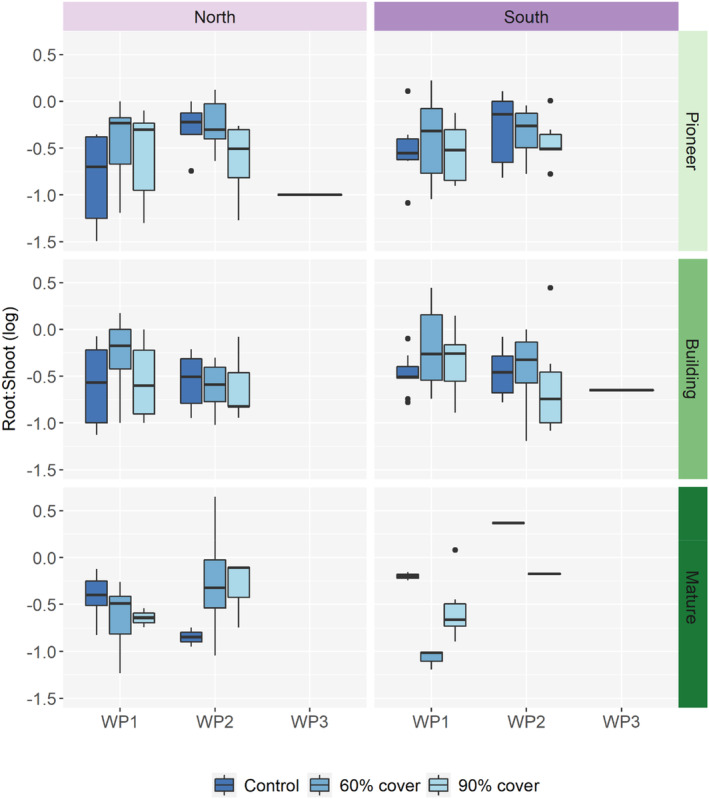
The logarithmic root to shoot ratio for seedlings emerged in the control (WP1), the mild drought (WP2) and the medium drought (WP3). The data is based on one seedling for each of the petri dishes that had emerged seedlings. Seedlings were harvested for the length and weight measurements 1 week after registered emergence. *N* = 206 seeds, 206 petri dishes.

**FIGURE A4:**
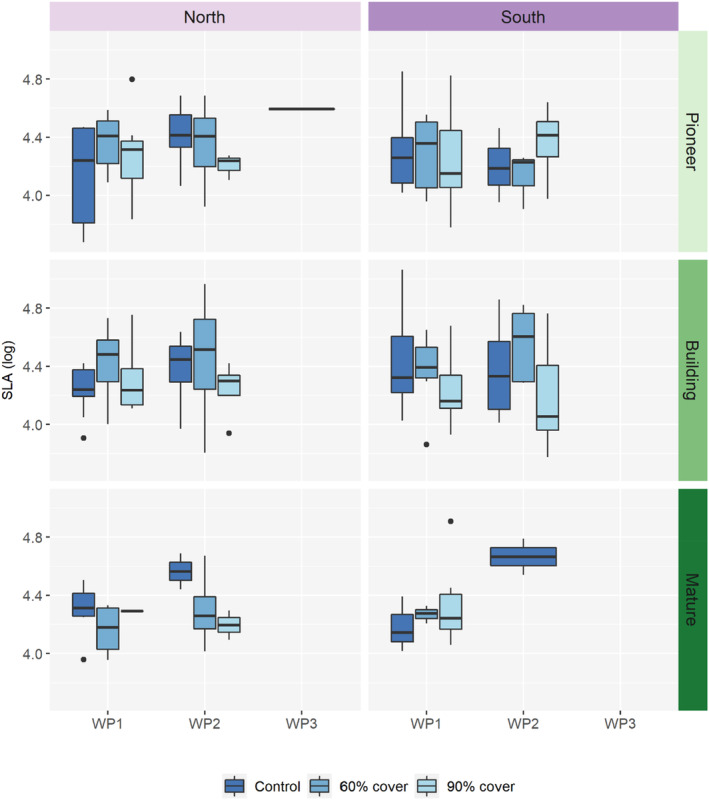
The logarithmic specific leaf area (SLA) for seedlings germinated in a control and the first and second level of drought. The seedlings were harvested for measurements 1 week after registered emergence. The seedlings emerged from seeds collected from a northern and southern population with three successional stages (pioneer, building, mature) and maternal drought treatments (control, 60% and 90% roof coverage) within.

Even though at least some *Calluna* seeds germinated under all water potential treatments, WP1, WP2, and WP3 (*n* = 3, WP3 is not reported on due to low sample size), were the only ones that fully developed into seedlings. Meanwhile, in WP4 and WP5, the seedlings stopped growing before they could be measured for seedling traits. Seedlings in WP2 grew shorter and thicker roots, resulting in a negative effect of water availability on SRL (Figure 5, see Figure A1 for pictures, post‐hoc test; WP1 vs. WP2: *p* < .001). This effect is especially prominent in the pioneer stage, reflected in a close to significant two‐way interaction between successional stage and the water availability treatment (Table A4), where the pioneer stage was the only one with a significantly lower SRL in WP2 compared to WP1 (Figure 5, post‐hoc test; subset pioneer stage; WP1 vs. WP2: *p* < .001). There was also a significant effect of population (Table A4), where the northern population had lower SRL when exposed to WP2 compared to WP1 (Figure 5, post‐hoc test; subset north; WP1 vs. WP2: *p* = <.0001). The root:shoot was less affected, but mature‐stage seedlings exposed to reduced water availability (WP2) allocated more biomass to roots than those developing with less water limitation (WP1) (Figure A3, post‐hoc test; subset mature; WP1 vs. WP2: *p* = .0036). Specific leaf area is not affected by origin of population, successional stage, maternal drought treatment, or reduced water availability during seed germination and seedling development (Figure A4, Table A4).

### 3.4 Effect of seed mass on germination and seedling growth

**FIGURE 6:**
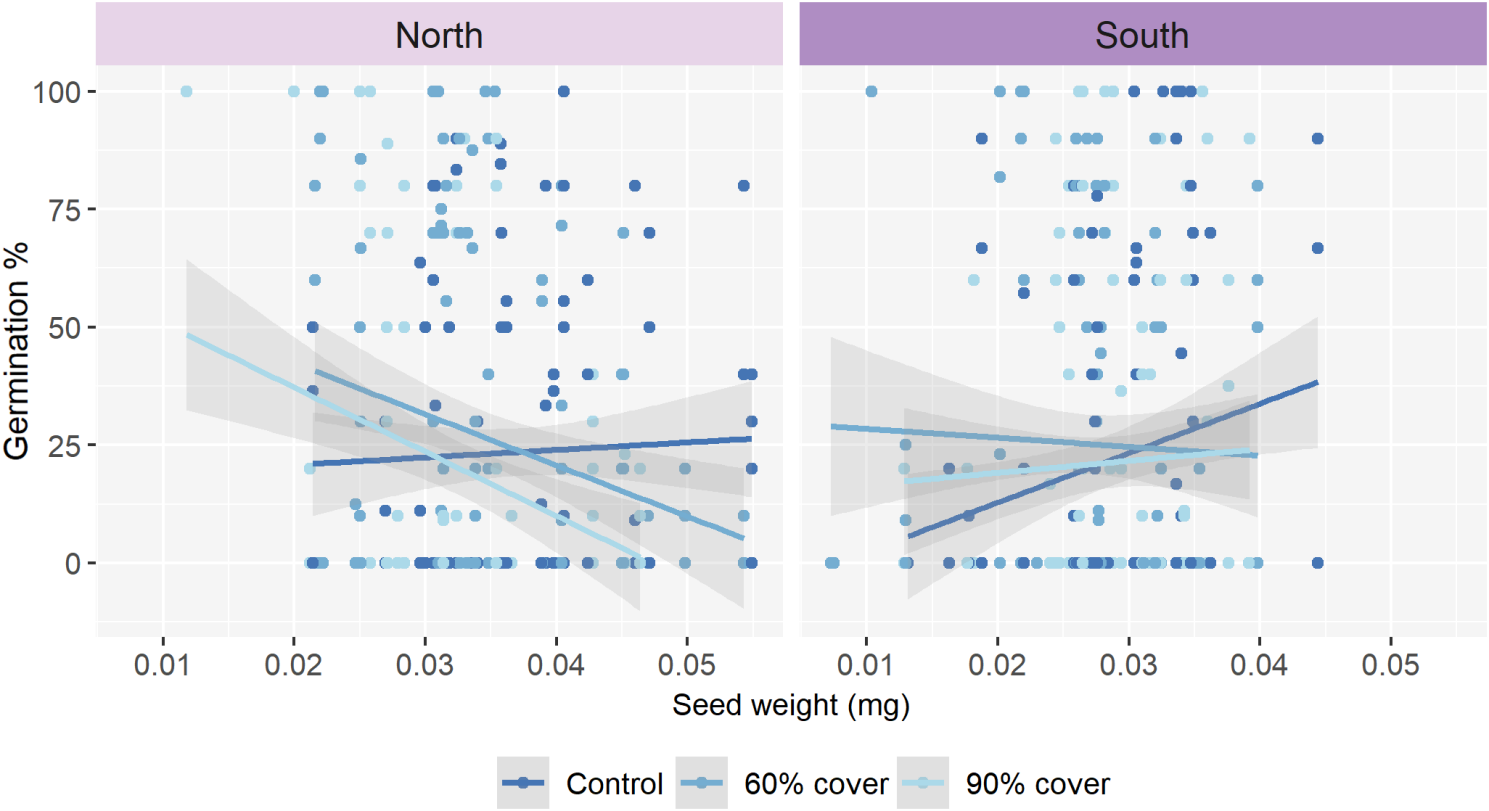
The effect of seed mass (mg) on the germination percentage for seeds from the maternal drought treatment (control, 60% and 90% roof coverage) within the northern and southern population. The seeds weight is on a per seed basis, based on samples of 50 seeds or sometimes less than. The germination was recorded during a 105‐day long lab germination experiment under controlled environments at 18°C with 8 h of growth light. *N* = 6530 seeds, 652 petri dishes.

There is a clear regional difference in the effect of seed mass on germination under ambient precipitation, being relatively unaffected in the northern population while increasing with increasing seed mass in the south (Figure 6, Table A2). Overall, the 60% and 90% roof coverage treatments decreased the slopes of the seed mass effects (Figure 6). However, due to differences in the ambient climate relationships, maternal drought exposure generally resulted in negative relationships between seed mass and germination in the north, whereas in the south, the positive relationship seen in the control plots disappeared (Figure 6).

After controlling for seed mass, the effects of population, successional stage from the model, and maternal drought treatment were no longer significant, indicating that these effects are largely controlled effects by variation in seed mass between the populations, successional stages, and maternal drought treatments (Table A2). In contrast, effects of water potential and its interactions generally increased after controlling for seed mass, indicating these effects are not regulated through variation in seed mass (Table A2).

**FIGURE A5:**
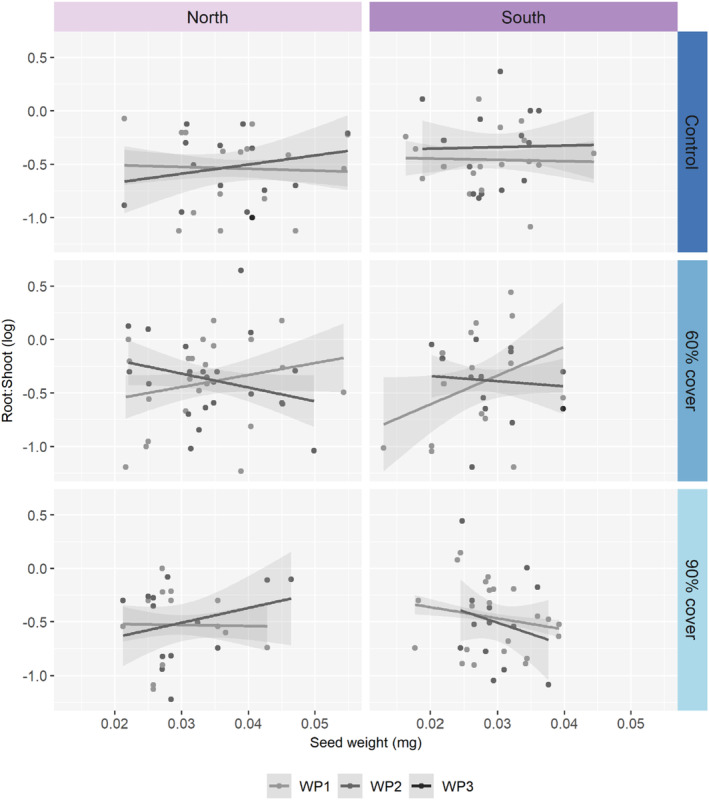
The effect of seed mass (mg) and reduced water availability (WP1, WP2 and WP3) on the logarithmic root to shoot ratio for seeds from the maternal drought treatment (control, 60% and 90% roof coverage) within the northern and southern population. The seeds weight is on a per seed basis, based on samples of 50 seeds. The seedlings were harvested for weight measurement 1 week after recorded emergence. *N* = 206 seeds, 206 petri dishes.

Seed mass did not affect root:shoot ratio (*p* = .975). Controlling for seed mass did not substantially change the output from the global model on root:shoot ratio, indicating seed mass does not affect seedling allocation to roots (Figure A5, Table A5).

## 4 DISCUSSION

Germination percentage plummeted in response to a modest reduction in water availability, and almost all aspects of the germination and seedling growth investigated were also affected. Under drought, seeds germinated more slowly, increasing both the time to 50% and time to maximum germination, and the seedling root length decreased while biomass allocation to roots increased. The only response not affected by reduced water availability was SLA. These effects were not constant, however, but varied between geographic regions and successional stages, and in response to maternal drought exposure. Whereas overall variation in germination rates and percentages between regions, successional stages, and maternal drought treatments were largely mediated by variation in seed mass, seed, and seedling drought sensitivity and growth was not. Complex interactions between these variables illustrate how *Calluna* from different regions and within different successional stages may differ in their ability to respond to and tolerate drought.

### 4.1 Seed mass varies across regions and with drought exposure

In line with expectation, the northern population has slightly heavier seeds (confirming P1, Table 1). Such population‐level differences in seed mass could simply reflect a trade‐off between seed mass and number of seeds (Grime, 2006; Grime & Pierce, 2012). However, this trade‐off was not evident in our data – as the northern *Calluna* population produced both heavier and more seeds (see Table 2 for number of seeds). As Notarnicola et al. (2023) noted, there has been mixed support for the seed mass / number trade‐off in the literature, where previous studies have found species to show negative, neutral, and positive relationships between seed size and number (Brancalion & Rodrigues, 2014; Guo et al., 2010; Lázaro & Larrinaga, 2018; Paul‐Victor & Turnbull, 2009) suggesting maternal investment in seeds is often co‐regulated by other selective pressures. Northern *Calluna* populations have been shown to have a reduced ability to revegetate through root sprouting (Nilsen et al., 2005; Velle & Vandvik, 2014). This suggests greater dependence on seeds for recruitment, and hence a potential driver of selection for larger maternal investment in seeds in the northern heathlands. Larger seeds have been found to be more densely packed with nutrients (Vaughton & Ramsey, 2001) and can thus have higher seedling establishment success (Ellison, 2001; Moles & Westoby, 2006; Notarnicola et al., 2023), and accordingly, we found that variation in seed mass between the northern and southern population did contribute to variation in germination rate and success. Overall, our findings thus support the prediction that northern *Calluna* plants invest more in seedling recruitment compared to the southern plants (both in terms of more seeds, larger seeds, and enhanced germination), reflecting a potentially adaptive response given their inability to reproduce vegetatively and, therefore, greater dependent on seeds for recruitment.

Following the same general argument, we also expected increased investment in seeds in later‐successional stages, as mature stands (Berdowski & Siepel, 1988; Hobbs & Gimingham, 1984; Meyer‐Grünefeldt et al., 2015; Miller & Miles, 1970) have also been shown to have reduced ability to revegetate through root sprouting. There was no overall difference in seed weight between successional stages (rejecting P2a, Table 1) and the mature stage also produced fewer seeds than earlier successional stages (see Table 2). The resulting lower reproductive output in mature‐stage heathlands may simply be linked to an age‐related reduction in fitness in the older late‐successional *Calluna* plants (cf. Haugum et al., 2021).

The maternal drought is causing a reduction in *Calluna* seed mass (rejecting P3, Table 1), which is seen specifically for the mature plants (confirming P2b, Table 1). Again, as argued above, a trade‐off between seed mass and numbers could have made up for this loss in fitness; however, the maternal drought‐exposed plants produced fewer seeds than under the ambient control (personal observations: Within the mature stage in both the north and south: Control = 1464, 60% cover = 1368, 90% cover = 1047). Instead and in parallel to the successional stage response, the maternal drought exposure seems to have resulted in reduced fitness of the plants leading to reduced seed quality and numbers and most pronounced in the most drought‐sensitive late‐successional stage (Haugum et al., 2021). A similar response has been found in *Wahlenbergia ceracea* (Notarnicola et al., 2023), where warming caused both a reduction in seed production and smaller seeds.

### 4.2 *Calluna* germination is sensitive to reduced water availability

Even a relatively moderate reduction in water availability during germination substantially affected germination rates and percentages. Surprisingly, seeds and seedlings of *Calluna*, a species with broad ecological tolerance and common in many dry heath and forest communities (Grant & Hunter, 1962; Halvorsen, 2016), were as sensitive to experimental drought conditions and were as negatively affected by experimental drought as alpine snowbed specialists *Sibbaldia procumbens* and *Veronica alpina*, studied in a comparable study by Gya et al. (2023). However, reduced water potentials significantly increased both the time to 50% and maximum germination and the viability test indicated that a majority of the seeds that did not germinate were still viable in the petri dishes at the end of the experiment. This suggests that the reduced germination under the reduced water availability in the lab experiment might reflect seed germination strategies to “avoid” seedling exposure to drought under field conditions, either through induced dormancy or strict germination requirements in response to drought, (see e.g., Vandvik et al., 2017 and references therein). In further support of this, *Calluna* seeds are known to have long lifespan in soil seed banks (Alday et al., 2023; Måren & Vandvik, 2009) and germination cueing linked to fire and land use (Vandvik et al., 2014) and climate (Spindelböck et al., 2013). In further support of a germination strategy‐related response, seed and seedling sensitivity to drought was not related to seed mass, suggesting physiological germination‐regulating responses rather than resource‐related effects. Such germination regulation in response to drought could allow *Calluna* to cue germination on times and places with relatively low drought risk, increasing the probability of survival of the sensitive seedlings.

The northern seeds had a higher germination percentage, which is in line with our prediction (P5, Table 1). The northern populations' overall higher germination percentage is expected in light of its lack of root sprouting (Nilsen et al., 2005; Velle & Vandvik, 2014). As the selection pressure on reproduction is different between the two populations, differences in the germination percentage would be expected. Even though we expected the northern population to have a higher germination percentage, we expected the southern population to have a broader germination niche; however, there were no evidence of this (P6, Table 1). Differences between populations' responses to drought have been observed in other populations of *Calluna* in Europe as well (Ibe et al., 2020; Meyer‐Grünefeldt et al., 2016). Meyer‐Grünefeldt et al. (2016) found southern and eastern European seedlings of *Calluna* to be more drought‐resistant compared to central European populations. Linking this to the southern populations climatic history of being more drought‐exposed (Meteorologisk Institutt, 2021), we assumed there would be a similar effect in this experiment. However, there were no indications of different responses between the populations looking at the germination niche.

The seeds from the mature successional stage were found to have a lower germination percentage compared to the younger stages, well aligned with our prediction (P7, Table 1). The generally lower germination in the mature stage was hypothesized to be due to a lower seed quality. We do know the older *Calluna* has a reduced ability to revegetate through root sprouting (Berdowski & Siepel, 1988; Hobbs & Gimingham, 1984; Meyer‐Grünefeldt et al., 2015; Miller & Miles, 1970; Nilsen et al., 2005; Schellenberg & Bergmeier, 2022) and has previously shown a greater sensitivity to drought (Haugum et al., 2021). This indicates an overall reduction in the plants performance with age, which was our baseline for the prediction of the older successional stages having a lower germination. We also found the maternal drought treatment (both 60% and 90% cover) to cause a wider germination niche, confirming P8 (Table 1), within the aboveground mature stage. The widened niche could be a maternal effect (Roach & Wulff, 1987), where the maternal plants' environment, in this case drought, has led to a more drought‐tolerant phenotype. However, the seemingly widened germination niche within the two older successional stages when exposed to the roof coverage could be due to the generally lower germination percentage at the lower water potentials, making it nearly impossible to conclude that this is a drought response found within the two older successional stages.

A greater seed mass had both a positive and negative effect on the germination percentage, partially rejecting our prediction (P4, Table 1). In the northern population, a slight positive effect was found for seeds from the control plot for the maternal drought treatment; however, it shifted for the 60% and 90% roof coverage, where a greater seed mass seems to have a more negative effect on the germination percentage. While the southern population exhibited a positive trend with a higher germination percentage for heavier seeds within the control in the maternal drought treatment. This trend flattened out for the offspring of the 60% and 90% drought‐exposed maternal generation, indicating an effect of the drought exposure during seed development. These results indicate that during ambient conditions, *Calluna* seeds from the southern population exhibit a positive correlation between a larger seed mass and increased germination. However, increased drought during seed production is altering the quality of the seeds, both reducing the mass and reducing especially larger seeds' ability to germinate. This effect is more prominent in the northern population, which is historically less prone to drought (Meteorologisk Institutt, 2021) and further, has previously been found to be less drought‐resilient (Haugum et al., 2021). For *Calluna*, previous studies find no effect of seed mass on germination along an altitudinal gradient in northern Spain (Vera, 1997). Previous research and our own results indicate *Calluna* have local adaptations in seed production and drought tolerance during germination.

### 4.3 Reduced water availability increases allocation to roots

Reduced water availability during germination had a major effect on the development of seedlings. Even if seeds germinated in all of the levels of reduced water availability, only WP1, WP2, and WP3 had fully developed seedlings. It is evident that the reduced water availability either delayed the development of the seedlings or caused the germinated seeds to die off before reaching the seedling stage. It is important to note that only two petri dishes had seedlings that emerged in WP3 compared to the 26 petri dishes that had germinated seeds. Seedling establishment and growth is a vulnerable stage during *Callunas'* life history (Meyer‐Grünefeldt et al., 2015; Schellenberg & Bergmeier, 2022), and our results suggest that drought exposure may further increase this vulnerability.

Reduced water availability increased *Callunas'* investment into the roots compared to the shoots, causing an increase in the root: shoot ratio, confirming parts of prediction P12 (Table 1). The resource uptake theory states that organs increasing the uptake of the most limiting resource for growth will have a higher allocation (Bloom et al., 1985). This strategy would increase the chances of survival and increase the fitness of the individual plant, and in this case by an increased investment into roots for a greater water uptake. While we expected to find an increase in SRL as a response to the reduced water availability, since longer roots would increase the surface area for a greater chance of water uptake (Liu & Stützel, 2004; Metcalfe et al., 2008); however, we found decreased SRL and shorter and thicker under drought (Personal observation; thickness of the root was not measured, but see Figure A1 for pictures). Both increasing (Comas et al., 2013) and decreasing (Zhou et al., 2018) SRL could be argued as a drought response, and there is not a very clear trend in the literature on how SRL in woody plants might respond to drought (Olmo et al., 2014). In a meta‐study by Zhou et al. (2018), they found a decrease in SRL as a response to drought at a global scale. Here they argued a lower SRL is an adaptation to an environment where water and nutrients would be limited for absorption, causing a slower growth rate, but a longer root lifespan. It could also be due to the symbioses with mycorrhizal fungi, which could cover the acquiring of nutrients for the host plant with a low SRL, compensating for reduced root length (see Wen et al., 2022).

The southern population had a slightly lower SRL compared to the northern population, rejecting prediction P9 (Table 1). The differences between the population's trait responses during drought could be linked to the populations' different climatic adaptations, which have been observed in *Calluna* elsewhere in Europe (Meyer‐Grünefeldt et al., 2016). The southern population within our experiment has been historically more subjected to drought and would be more adapted to this. This could be expressed through more plastic responses to drought during seedling development. The southern population also showed more of an increase in the root:shoot ratio with a greater seed mass in comparison to the northern, well aligned with prediction P4 (Table 1). Larger seeds have previously been found to increase the weight and survival of *Calluna* seedlings in northern Spain (Vera, 1997), which in combination with our results might suggest that there are local adaptations in *Calluna*.

Even though the low water availability caused a higher allocation to roots, the SLA was not affected. From a previous meta‐analysis, drought was found to cause seedlings to produce smaller and thicker leaves (Wellstein et al., 2017). This was also expected during our experiment with reduced water availability (P12, Table 1). Our analysis was done on cotyledons, which has been found to have a greater response in SLA compared to true leaves (Nesheim‐Hauge, 2018), indicating a lack of response in SLA in *Calluna*. However, fully developed seedling could have shown trends in SLA that we did not get at such an early stage. There was also no evidence to support our predictions of seeds from the mature successional stage or the maternal drought treatment growing a lower root:shoot ratio or SRL, or a greater SLA (rejecting P10 and P11, Table 1). A higher sampling effort and collecting true seedlings could potentially have created clearer trends and changed the results. More research is needed to fully understand *Calluna* seedlings' responses to drought.

## 5 CONSERVATION IMPLICATIONS UNDER A CHANGING CLIMATE

Our results add another layer of knowledge to how *Calluna* populations, successional stages, and individual plants respond to drought. The northern population had an overall higher germination percentage and wider germination niche in response to drought but exhibited no traits or responses that confer drought tolerance in its seedling stage. Based on these results, it is fair to assume the northern population will face greater threats as drought events are becoming more frequent, in comparison to the historically more drought‐prone southern population.

The mature successional stage was found to be more sensitive to drought than younger successional stages, both in the field and the lab. If exposed to drought, these plants produced smaller seeds that germinated less. Due to the abandonment of traditional low‐intensity land use, coastal heathlands are currently red‐listed throughout their range, with mature and degraded *Calluna* dominating (Hovstad et al., 2018). Considering the increasing events of drought, we could expect to see a decrease in seed mass and germination percentage as drought is turning into the new normal. The effect of this would be a reduced possibility for mature stands to reproduce, both through their already reduced ability to do vegetative root sprouting (Berdowski & Siepel, 1988; Hobbs & Gimingham, 1984; Meyer‐Grünefeldt et al., 2015; Miller & Miles, 1970), but also their reduced investment into its seeds during drought, further threatening an already vulnerable and red‐listed ecosystem.

This research is a contribution to our understanding of how drought affects the germination and early development of *Calluna*. We found *Calluna* to be surprisingly sensitive to drought during germination and seedling stage. And even though we found the seedlings to have drought‐adapted root traits, there was a low emergence of seedlings under drought conditions even if the seeds germinated. This knowledge should be taken into consideration for heathland management in a changing climate. Well‐managed heathlands dominated by younger successional stages are more resilient to drought, and using fire as a tool to bring back healthy heathlands is a needed practice (Bargmann, Måren & Vandvik, 2014; Haugum et al., 2021). Seedling recruitment mainly happens after fire management and burning in mosaic could potentially reduce the negative impact of low germination and recruitment in a drought year. However, further research is needed to fully understand the impact of drought on seedling recruitment and survival in *Calluna*. Further research should also look further into the traits of *Calluna* seedlings throughout the development during drought conditions.

## AUTHOR CONTRIBUTIONS

**Kristine Birkeli:** Conceptualization (equal); data curation (lead); formal analysis (lead); investigation (lead); methodology (equal); writing – original draft (lead); writing – review and editing (equal). **Ragnhild Gya:** Methodology (lead); writing – original draft (supporting); writing – review and editing (supporting). **Siri Vatsø Haugum:** Conceptualization (equal); methodology (equal); supervision (equal); writing – original draft (supporting); writing – review and editing (supporting). **Liv Guri Velle:** Conceptualization (equal); methodology (equal); project administration (lead); writing – original draft (supporting); writing – review and editing (supporting). **Vigdis Vandvik:** Conceptualization (equal); methodology (equal); project administration (lead); supervision (lead); writing – original draft (supporting); writing – review and editing (equal).

## FUNDING INFORMATION

This project is financially supported by the Norwegian Research Council, under the LandPress project and the Recite project.

## CONFLICT OF INTEREST STATEMENT

The authors declare no conflict of interest.

## Data Availability

The data, script for data management and analysis is available at the Open Science Framework (https://doi.org/10.17605/OSF.IO/NU7MV).

## APPENDIX 1

**TABLE A1:** Anova output for the effect of population (north and south), successional stage (pioneer, building, mature), and maternal drought treatment (ambient control, 60% and 90% roof coverage) on seed mass with chi‐square (Chisq), degrees of freedom (df) and the *p*‐value (Pr(>Chisq)).

Variable	Chisq	df	Pr(>Chisq)
Population (*P*)	3.473	1	.062
Successional stage (SS)	0.457	2	.796
Maternal drought treatment (MDT)	1.521	2	.467
*P*:SS	1.021	2	.600
*P*:MDT	4.775	2	.092
SS:MDT	3.460	4	.484
*P*:SS:MDT	**18.112**	**4**	**.001**

*Note*: Significant values marked in bold (*p* < .05).

**TABLE A2:** Anova output for the effect of population (north and south), successional stage (pioneer, building, mature), maternal drought treatment (ambient control, 60% and 90% roof coverage), and reduced water availability (WP1–WP5) on the germination percentage with a binomial distribution and the germination percentage using the residuals from seed mass effect on germination percentage.

Model	Germination percentage			Germination percentage (using residuals from seed mass)		
Variable	Chisq	df	Pr(>Chisq)	Chisq	df	Pr(>Chisq)
Population (*P*)	**8.330**	**3**	**.039658**	0.400	1	.527
Successional stage (SS)	**53.857**	**2**	**<.0001**	1.379	2	.502
Maternal drought treatment (MDT)	7.388	3	.0605	0.099	2	.952
Reduced water availability (WP)	**596.067**	**8**	**<.0001**	**1778.095**	**4**	**<.0001**
*P*:SS	**7.794**	**2**	**.0203**	0.792	2	.673
*P*:MDT	**11.442**	**3**	**.0095**	0.083	2	.959
SS:MDT	**9.852**	**4**	**.043**	0.205	4	.995
*P*:WP	**20.246**	**5**	**.0011**	7.324	4	.120
SS:WP	**23.537**	**8**	**.0027**	**125.333**	**8**	**<.0001**
MDT:WP	13.418	9	.145	6.862	8	.552
*P*:SS:MDT	5.605	4	.231	0.864	4	.930
*P*:SS:WP	3.322	8	.913	**27.705**	**8**	**.0005**
*P*:MDT:WP	8.654	8	.372	8.453	8	.391
SS:MDT:WP	8.667	16	.926	47.008	16	**<.0001**
*P*:SS:MDT:WP	7.557	16	.961	22.777	16	.120

*Note*: Here with chi‐square (Chisq), degrees of freedom (df) and the *p*‐value (Pr(>Chisq)). Significant values marked in bold (*p* < .05).

**TABLE A3:** Anova output for the effect of population (north and south), successional stage (pioneer, building, mature), maternal drought treatment (ambient control, 60% and 90% roof coverage), and reduced water availability (WP1–WP5) on time to 50% germination (*T*_50_) and maximum germination (*T*_max_).

Model	*T*_50_			*T*_max_		
Variable	Chisq	df	Pr(>Chisq)	Chisq	df	Pr(>Chisq)
Population (*P*)	1.506	1	.219	0.651	1	.420
Successional stage (SS)	0.331	2	.847	0.679	2	.712
Maternal drought treatment (MDT)	**6.696**	**2**	**.035**	1.294	2	.524
Reduced water availability (WP)	**489.793**	**4**	**<.0001**	**136.379**	**4**	**<.0001**
*P*:SS	0.307	2	.858	0.765	2	.682
*P*:MDT	1.850	2	.397	1.862	2	.394
SS:MDT	8.488	4	.075	4.426	4	.351
*P*:WP	**20.195**	**3**	**.00015**	**24.950**	**3**	**<.0001**
SS:WP	**84.365**	**7**	**<.0001**	**34.450**	**7**	**<.0001**
MDT:WP	**42.320**	**5**	**<.0001**	**25.894**	**5**	**<.0001**
*P*:SS:MDT	1.105	4	.894	0.244	4	.993
*P*:SS:WP	5.012	3	.171	6.205	3	.102
*P*:MDT:WP	1.548	4	.818	9.340	4	.0531
SS:MDT:WP	**28.527**	**7**	**.00018**	**16.969**	**7**	**.018**
*P*:SS:MDT:WP	8.191	5	.146	**14.055**	**5**	**.015**

*Note*: Here with chi‐square (Chisq), degrees of freedom (df) and the *p*‐value (Pr(>Chisq)). Significant values marked in bold (*p* < .05).

**TABLE A4:** Anova output for the effect of population (north and south), successional stage (pioneer, building, mature), maternal drought treatment (ambient control, 60% and 90% roof coverage), and reduced water availability (WP1–WP5) on the specific root length (SRL) and specific leaf area (SLA) with chi‐squared (Chisq), degrees of freedom (df) and the *p*‐value (Pr(>Chisq)).

Model	SRL			SLA		
Variable	Chisq	df	Pr(>Chisq)	Chisq	df	Pr(>Chisq)
Population (*P*)	**3.866**	**1**	**.049**	0.002	1	.966
Successional stage (SS)	4.137	2	.126	1.632	2	.442
Maternal drought treatment (MDT)	0.066	2	.968	2.599	2	.273
Reduced water availability (WP)	**55.933**	**2**	**<.0001**	2.470	2	.291
*P*:SS	0.208	2	.901	0.385	2	.825
*P*:MDT	1.081	2	.582	0.636	2	.728
SS:MDT	2.493	4	.646	3.737	4	.443
*P*:WP	0.720	2	.698	0.425	1	.514
SS:WP	5.459	2	.065	2.386	2	.303
MDT:WP	3.132	2	.209	0.647	2	.724
*P*:SS:MDT	6.876	4	.143	3.450	4	.486
*P*:SS:WP	2.714	2	.257	2.324	2	.313
*P*:MDT:WP	0.087	2	.957	3.815	2	.148
SS:MDT:WP	5.767	4	.217	2.202	4	.699
*P*:SS:MDT:WP	1.679	3	.642	1.077	2	.584

*Note*: Significant values marked in bold (*p* < .05).

**TABLE A5:** Anova output for the effect of population (north and south), successional stage (pioneer, building, mature), maternal drought treatment (ambient control, 60% and 90% roof coverage), and reduced water availability (WP1–WP5) on root:shoot and root:shoot using the residuals from seed mass effect on root:shoot.

Model	Root:shoot			Root:shoot (using residuals from seed mass)		
Variable	Chisq	df	Pr(>Chisq)	Chisq	df	Pr(>Chisq)
Population (*P*)	2.588	1	.108	1.389	1	.239
Successional stage (SS)	0.167	2	.920	0.822	2	.663
Maternal drought treatment (MDT)	3.829	2	.147	3.147	2	.207
Reduced water availability (WP)	2.400	2	.301	3.571	2	.168
*P*:SS	0.071	2	.965	0.686	2	.710
*P*:MDT	1.417	2	.492	0.568	2	.753
SS:MDT	5.524	4	.238	5.157	4	.272
*P*:WP	0.174	2	.917	0.127	2	.938
SS:WP	**8.449**	**2**	**.015**	**8.198**	**2**	**.017**
MDT:WP	2.327	2	.312	1.325	2	.516
*P*:SS:MDT	3.102	4	.541	4.279	4	.370
*P*:SS:WP	1.940	2	.379	1.707	2	.426
*P*:MDT:WP	0.047	2	.977	0.113	2	.945
SS:MDT:WP	6.905	4	.141	7.012	4	.135
*P*:SS:MDT:WP	3.493	3	.322	2.255	3	.521

*Note*: Here with chi‐square (Chisq), degrees of freedom (df) and the *p*‐value (Pr(>Chisq)). Significant values marked in bold (*p* < .05).
